# A Non-Invasive Pathogen‑Responsive RUBY Reporter Enables Visible Monitoring of *PR1* Activation

**DOI:** 10.17912/micropub.biology.002174

**Published:** 2026-08-11

**Authors:** Lola O. McMullan, Joseph S. Taylor, Regina Hanlon, Alex T. Harris, Joel L. Shuman, David G. Schmale III, Bastiaan O. R. Bargmann

**Affiliations:** 1 School of Plant and Environmental Sciences, Virginia Polytechnic Institute and State University, Blacksburg, VA, US

## Abstract

We report a non‑invasive, pathogen‑responsive reporter system in Arabidopsis thaliana generated by placing the PR1 (AT2G14610) promoter upstream of the betalain‑producing RUBY cassette. Two independent transgenic lines showed robust, dose‑dependent red pigmentation accumulation in response to flg22 (a flagellin-derived peptide) and salicylic acid (a defense-signaling molecule that induces *PR1* ) in both seedling and adult tissues. This reporter provides a simple, low‑cost visual readout of defense activation without the need for destructive sampling or specialized instrumentation. Seeds have been deposited at ABRC, and the plasmid has been made available through Addgene.

**Figure 1. Pathogen‑responsive PR1::RUBY reporter activation in Arabidopsis:** **A.** Schematic of the *PR1::RUBY* construct. A 1,334 bp fragment of the Arabidopsis *PR1* promoter (AT2G14610) was cloned into the *Spe* I site of pHDE‑RUBY, placing the betalain biosynthetic cassette (CYP76AD1–2A–DODA–2A–glucosyltransferase) under pathogen‑responsive transcriptional control. **B.** One‑week‑old seedlings of two independent transgenic lines (1.6 and 2.4) grown in 24‑well plates (five seedlings per well) showed dose‑dependent betalain accumulation after 48 h treatment with flg22 (0–5 µM) or salicylic acid (SA) (0–100 µM). Asterisks indicate wells shown in close-up in C. **C.** Close-up images of the control, 5 µM flg22, and 100 µM SA treatment of line 2.4. **D.** Four‑week‑old soil‑grown plants (line 2.4) 48 h after treatment with 5 µL droplets of mock solution (0.05% Silwet), 1 µM flg22, or 50 µM SA to each leaf. These results are representative of multiple experiments.

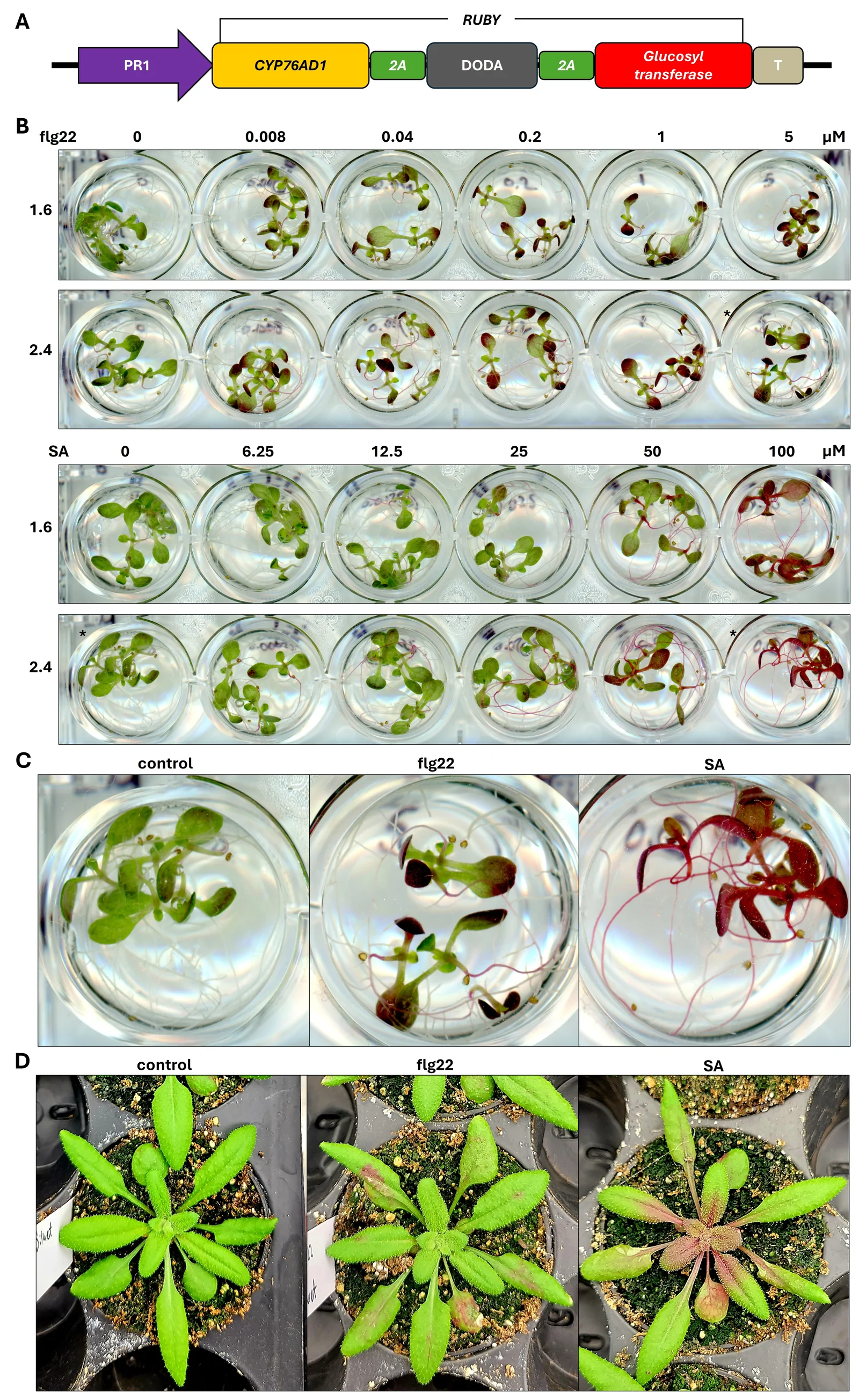



## Description

Plant immune responses are highly dynamic, spatially heterogeneous, and often difficult to monitor without destructive sampling or specialized equipment. The *Arabidopsis thaliana* *PATHOGENESIS‑RELATED 1 (PR1,* AT2G14610 *)* promoter is one of the most widely used molecular markers of salicylic acid (SA)–dependent defense activation (Ward et al., 1991), yet most *PR1* reporters rely on fluorescent or enzymatic outputs that require microscopy, substrate addition, and/or tissue disruption. To provide a simple, low‑cost, and non‑invasive alternative, we engineered a visible pathogen‑responsive reporter by placing a 1,334 bp *PR1* promoter fragment upstream of the RUBY betalain biosynthetic cassette (He et al., 2020) ( Figure 1A ). RUBY produces red betalain pigments that accumulate without additional substrates and are easily detected by eye, making it an attractive reporter for rapid screening, phenotyping, and educational applications.

We cloned the *PR1* promoter into pHDE‑RUBY, generating a *PR1::RUBY* construct that links immune activation to betalain production. Using *Agrobacterium tumefaciens* GV3101, we transformed *Arabidopsis thaliana* Col‑0 and selected two independent single‑locus lines (lines 1.6 and 2.4; single locus status was determined by examining the segregation ratio of hygromycin resistance in the T2 generation). Both lines exhibited robust and reproducible reporter activation in response to flg22 (a flagellin-derived peptide) and SA. In a 24‑well plate assay, one‑week‑old seedlings treated for 48 h with flg22 (0–5 µM) (Felix et al., 1999) and SA (0–100 µM) showed clear, dose‑dependent accumulation of red pigmentation, with no obvious signal in untreated controls ( Figure 1B- C). Line 2.4 displayed slightly higher sensitivity, but both lines responded similarly across the full concentration range. SA elicited the strongest overall activation, consistent with its role as a central regulator of *PR1* expression (Chen et al., 2020).

To test reporter performance in mature tissues, we applied 5 µL droplets of mock solution (0.05% Silwet only), 1 µM flg22, or 50 µM SA to individual leaves of four‑week‑old soil‑grown plants. After 48 h, flg22 and SA treatments induced visible betalain accumulation, whereas mock‑treated leaves remained green ( Figure 1D ). These results demonstrate that *PR1::RUBY* reliably reports immune activation in both seedlings and adult plants, and that betalain accumulation provides a clear, easily interpretable visual output.

This reporter system offers several advantages for the plant research community. First, it enables rapid, non‑destructive monitoring of defense activation without specialized imaging equipment. Second, it is suitable for high‑throughput screening in multi‑well formats. Third, it provides an intuitive visual readout that is accessible for research, teaching, and outreach. Future work will evaluate reporter activation during infection with well-characterized Arabidopsis pathogens, such as *Pseudomonas syringae* and *Hyaloperonospora arabidopsidis* (Cameron et al., 1999; Marco et al., 2014). Because the RUBY cassette functions across diverse plant species, the construct may also be adaptable beyond Arabidopsis. To our knowledge, this is the first report of *PR1::RUBY* reporter in use, although we did find mention of such a construct on an International Genetically Engineered Machine (iGEM) project website ( https://2021.igem.org/Team:Duesseldorf/Proof_Of_Concept ). Future studies incorporating quantitative image analysis or spectrophotometric assays of betalain accumulation will further enable rigorous characterization of reporter sensitivity, dose response, and reproducibility. We envisage its application as a sentinel phytosensor for the presence of plant pathogens. To facilitate broad use, the *PR1::RUBY* plasmid was deposited at Addgene, and the transgenic Arabidopsis lines were made available through the Arabidopsis Biological Resource Center (ABRC).

## Methods

**Construct assembly**

A 1,334 bp fragment upstream of the *Arabidopsis thaliana PR1* coding sequence was amplified using primers *proPR1_Spe* I_Fwd TGACTG *ACTAGT* ACGTAATAATATCCTATGGTGTC and *proPR1_Spe* I_Rev TGACTG *ACTAGT* CATTTTTCTAAGTTGATAATGGTTATTG (based on (Pape et al., 2010)) and cloned into the *Spe* I site of pHDE‑RUBY (He et al., 2020), placing the RUBY betalain biosynthetic cassette under *PR1* promoter control. The pHDE_PR1::RUBY vector is available through Addgene ( https://www.addgene.org/255430/ : Plasmid #255430).

**Plant transformation and growth conditions**

The construct was transformed into *A. thaliana* Col‑0 using *Agrobacterium tumefaciens* GV3101 via floral dip. T1 plants were selected, and two independent single‑locus lines (1.6 and 2.4) were advanced for characterization. Seeds for both lines are available through the ABRC stock center ( https://abrc.osu.edu/ ; CS74214 - pHDE_PR1::RUBY 1.6 and CS74215 - pHDE_PR1::RUBY 2.4). Seedlings were grown in ½ MS medium (2.2 g/L Murashige and Skoog salts) supplemented with 1% (w/v) sucrose, incubated in a plant growth chamber (Percival, Perry, IA, USA) at 22 °C with an 18 h light/6 h dark regime at 75 µmol m ^−2^ s ^−1^ PAR. Soil-grown plants were incubated in a plant growth chamber (Conviron, Pembina, ND, USA) at 22 °C with an 18 h light/6 h dark regime at 150 µmol m ^−2^ s ^−1^ PAR.

**Elicitation assays**

Five seedlings were grown in each well of a 24‑well plate containing 500 µL liquid ½ MS + sucrose medium. After one week, medium in the wells was replaced with 500 µL liquid ½ MS + sucrose supplemented with flg22 (0, 0.008, 0.04, 0.2, 1, or 5 µM) or salicylic acid (0, 6.25, 12.5, 25, 50, or 100 µM). Plates were incubated for 48 h and imaged using a flatbed document scanner. Four‑week‑old soil‑grown plants were treated by applying 5 µL droplets of 0.05% (v/v) Silwet (mock), Silwet plus 1 µM flg22, or Silwet plus 50 µM SA to each leaf. Plants were imaged after 48 h using a digital camera.
